# Determination of sequence and absolute configuration of peptide amino acids by HPLC–MS/CD-based detection of liberated N-terminus phenylthiohydantoin amino acids

**DOI:** 10.1038/s41598-022-14205-x

**Published:** 2022-06-18

**Authors:** Dongyup Hahn, Weihong Wang, Hyukjae Choi, Heonjoong Kang

**Affiliations:** 1 School of Food Science and Biotechnology & Department of Integrative Biotechnology, Kyungpook National University, Daegu, 41566 South Korea; 2 Laboratory of Marine Drugs, School of Earth and Environmental Sciences, Seoul National University, NS-80, 1 Gwanak-ro, Gwanak-gu, Seoul, 08826 South Korea; 3 Research Institute of Oceanography, Seoul National University, Seoul, 08826 South Korea; 4 College of Pharmacy, Yeungnam University, 280 Daehak-ro, Gyeongsan-si, Gyeongsangbuk-do 38541 South Korea; 5 Interdisciplinary Graduate Program in Genetic Engineering, Seoul National University, NS-80, Seoul, 08826 South Korea

**Keywords:** Peptides, Bioanalytical chemistry, Circular dichroism, Mass spectrometry, Peptides, Stereochemistry, Structure elucidation

## Abstract

We report a method for the simultaneous determination of the sequence and absolute configuration of peptide amino acids using a combination of Edman degradation and HPLC–MS/CD. Phenylthiohydantoin (PTH) derivatives of 20 pairs of standard d- and l-amino acids were synthesized by the Edman reaction. The CD spectra of the derivatives revealed that each pair of the PTH derivatives exhibited the absorption with opposite signs at around 270 nm. These standard PTH derivatives showed well-resolved resolution without interference from byproducts in the ion chromatogram and clear positive/negative CD absorptions when subjected on a reversed phase HPLC–MS system coupled with a CD-2095 HPLC detector. This method was applied for the detection of a synthetic pentapeptide and a natural depsipeptide (halicylindramide C). The sequence and configuration of the pentapeptide and up to eight residues of halicylindramide C were successfully analyzed by this method. The amino acid configuration of the pentapeptide was also determined successfully by subjecting its acid hydrolysates to the Edman reaction followed by HPLC–MS/CD.

## Introduction

Chirality, a fundamental attribute of nature, has been found to be ubiquitous; it plays a pivotal role in biochemical environments^1^. Since the first report on ammonium tartrate enantiomers by Pasteur in 1858^2^, the stereochemistry of biomolecules has been a significant topic, as the construction of chiral interfaces and their effect on the behavior of various substances crucially affect various biological and physiological processes^1,3^. As with other biomolecules synthesized in biological systems, the stereochemistry and chiral recognition of proteins and peptides are strongly affected by the stereochemistry of their amino acid building blocks. The chirality of the constituent amino acids determines the three-dimensional structures of proteins and peptides, and the three-dimensional structure determines not only the binding properties of the molecule to receptors but also the structural resistance of the molecule to enzymatic degradation^4,5^.

Amino acids, except for glycine (Gly) the only achiral amino acid, in living organisms were long believed to exist solely as the l-configuration, as proteinogenic amino acids^6^. However, it is now well-known that both d- and l-amino acids exist in natural molecules. d-amino acid residues were first discovered in peptidoglycans within bacterial cell walls^7–9^ and peptidic antibiotics^7^, followed by the discovery of d-amino acid residues in amphibians^10,11^, spiders^12^, marine sponges^13^, mollusks^14,15^, insects^16^, crustaceans^17^, mammals^18,19^, and humans^20,21^. Biologically active peptides containing d-amino acids including didemnin B^22^, palau’amide^23^, cyclotheonamides^24^, and halicylindramides^13^ have been reported to have various therapeutic potentials.

To investigate the biomedical potential of natural peptides, it is crucial but challenging to determine the sequences and configurations of the constituent amino acids. The configurations of peptide amino acids have generally been determined by acid hydrolysis, diastereomeric derivatization, and chromatographic separation, including Marfey’s methods^25,26^ and the use of O-phthaldialdehyde and *N*-acetyl-l-cysteine^27^. However, because these methods do not provide information about the peptide sequences, they cannot be used to determine the positions of d-amino acids in peptides.

Edman degradation is widely used in the sequence analysis of peptides^28–30^ (Fig. SI1). In Edman degradation, the N-terminus amino acid is sequentially released from the peptide as a PTH derivative, and the sequentially produced PTH derivatives can be identified by using chromatographic techniques or electrophoresis^31,32^. The Edman reagents have been modified to enhance the analytical sensitivity of the method; however, the configuration of the amino acid residues cannot be determined using the classical Edman degradation method^33–37^.

In order to simultaneously determine the sequences and configurations of constituent amino acids in peptides, derivatization methods with chiral Edman reagents^38,39^ and analysis of enantiomeric Edman products by chiral chromatography^40^ have been suggested. These methods rely on chromatographic separation to determine the amino acid configurations, requiring laborious reaction steps, multiple separation steps, and, in some cases, chiral HPLC columns.

Circular dichroism (CD) spectroscopy is a convenient method for evaluating molecular optical activity; it has been used to determine the absolute configurations of chiral compounds containing carbonyl or phenyl moieties. Conjugated chromophores around a chiral center cause optical activity and CD absorption. Aromatic amino acids, such as Phe, Trp, and Tyr, show strong CD absorption between 250 and 300 nm^41–43^, while the other amino acids show no or only weak CD absorption in this region. There have been various attempts to resolve the sensitivity issue by employing biphenyl chromophores so that the amino acid derivatives could be detected using CD instruments^6,44–49^; however, only three amino acids could be detected during a measurement cycle.

In this study, we report an analytical method for simultaneously determining the sequence and absolute configurations of peptide amino acids, using a combination of the Edman reaction and HPLC–MS/CD. A PTH derivative of the amino acid produced by the Edman reaction contains a chromophore next to a chiral center retained from the original amino acid. The CD absorption of PTH-amino acid was used to determine the absolute configurations of amino acids. To confirm the accuracy of the method, analyses of a commercial pentapeptide and a natural depsipeptide, halicylindramide C, were performed.

## Results and discussion

### CD spectra of PTH derivatives of standard amino acids

**Figure 1:**
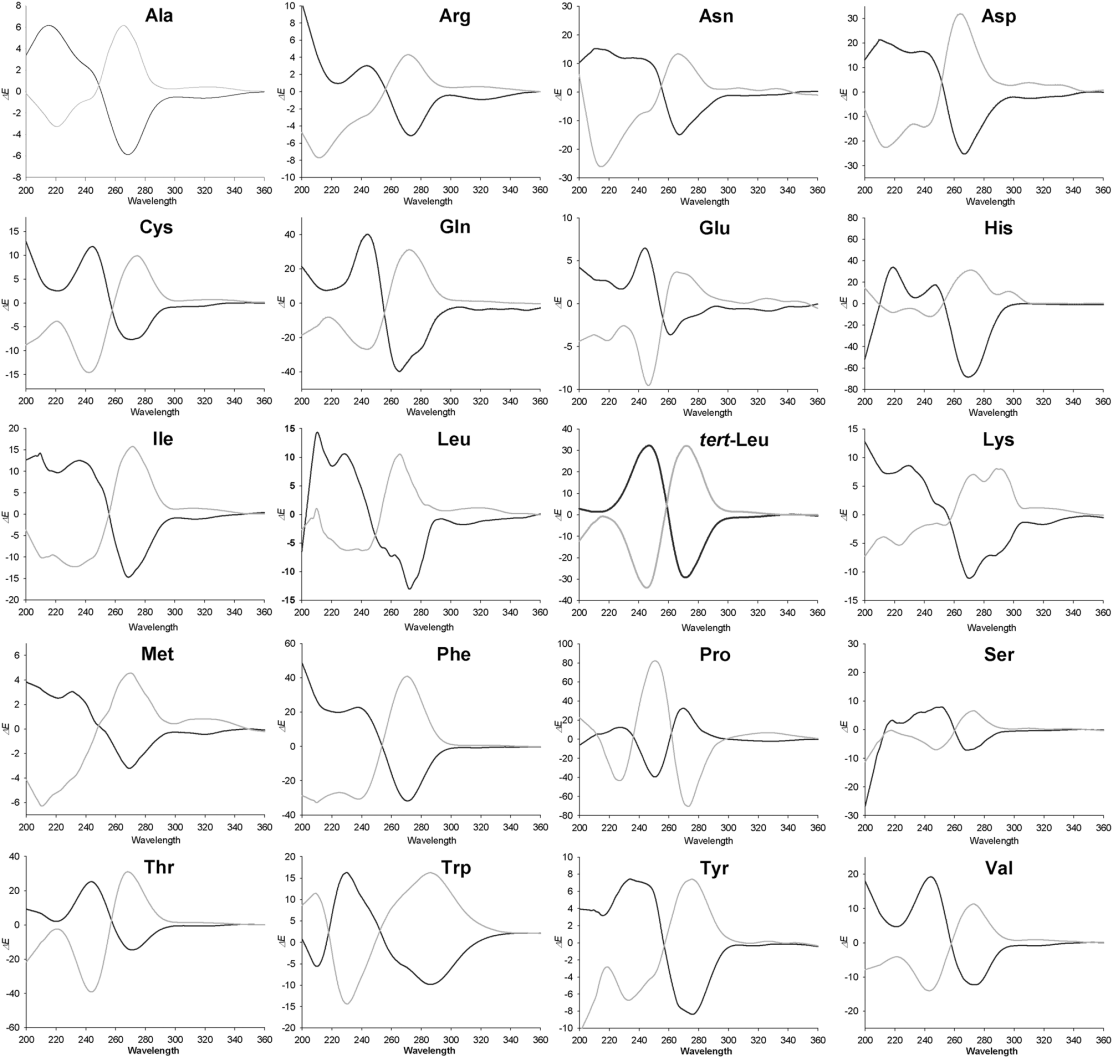
CD spectra of 1 µM of PTH derivatives of 20 pairs of amino acids (d-amino acid spectra are dark, l-amino acid light) in methanol.

PTH-amino acids contain a chiral center derived from the peptide amino acid. The chirality has been conserved through the Edman reaction by using an aprotic Lewis acid and hydrogen chloride-methanol (HCl–MeOH). The chiral center of a PTH derivative exists in the five-membered ring with a chromophore. Chirality, therefore, induces optical activity that is detectable with a CD spectrophotometer. We synthesized PTH derivatives of 19 common amino acids and *tert*-Leu and recorded their CD spectra. As shown in Fig. 1, PTH derivatives of d- and l-amino acids exhibited opposite-sign CD absorptions at 270 nm. PTH-l-Pro showed a negative CD absorption at 270 nm, while the remaining PTH derivatives of l-amino acids showed positive CD absorption. The pyrrolidine ring of Pro induces ring strain on its PTH derivative, which may be responsible for the distinctive CD absorption. It should be noted that excess phenyl isothiocyanate (PITC) will react with EtOH and the resulting byproduct may interfere with the CD absorption of PTH-amino acids (Fig. S2). In order to minimize byproduct generation and obtain accurate CD spectra of PTH-amino acids, a 1:1 ratio of PITC–amino acid was used for the synthesis of PTH derivatives of standard amino acids.

### Separation of PTH-amino acids on HPLC–MS/CD

The PTH derivatives of standard amino acids were separated through reverse phase (RP) HPLC–MS/CD. Twenty pairs of PTH-amino acids were separated on a C18 HPLC column with step gradient elution for 50 min. PTH-amino acids were identified by retention time and *m/z* value of ion peaks (Table 1). Carboxylic acids in Asp and Glu were esterified through the conversion step and the corresponding PTH derivatives were detected with *m/z* values of 265 and 279, respectively. The byproducts and impurities of the Edman reaction were also detected on the HPLC–MS ion chromatogram; however, those peaks detected on the CD-2095 detector were selectively analyzed. The major byproduct peak was eluted at the retention time of 19.5 min with the *m/z* value of 228, and identified as diethoxy(phenylamino)methanethiol produced from EtOH and PITC in the coupling solution (Fig. S2). Because the major byproduct did not overlap with PTH-amino acid derivatives on the HPLC chromatogram and did not exhibit any CD absorption, PTH-amino acid CD absorptions could be observed without interruption.

**Table 1:** Identification of PTH derivatives based on their retention times, *m/z* values, and CD absorption at 270 nm.

Amino acid	*m/z* values of PTH derivative	Retention time of PTH derivative (min)	CD absorption of PTH derivative	
			l-amino acid	d-amino acid
Ala	207	14.60	+	−
Arg	292	3.52	+	−
Asn	250	4.22	+	−
Asp	265	16.67	+	−
Cys	223	4.76	+	−
Gln	264	6.98	+	−
Glu	279	23.42	+	−
Gly	193	6.18		
His	273	1.81	+	−
Ile	249	42.60	+	−
Leu	249	43.78	+	−
*tert*-Leu	249	43.11	+	−
Lys	399	46.08	+	−
Met	267	29.98	+	−
Phe	283	44.98	+	−
Pro	233	26.33	−	+
Ser	223	4.82	+	−
Thr	237	12.49	+	−
Trp	322	44.30	+	−
Tyr	299	21.82	+	−
Val	235	29.01	+	−

### Sequence and configuration analysis of a commercial peptide

**Figure 2:**
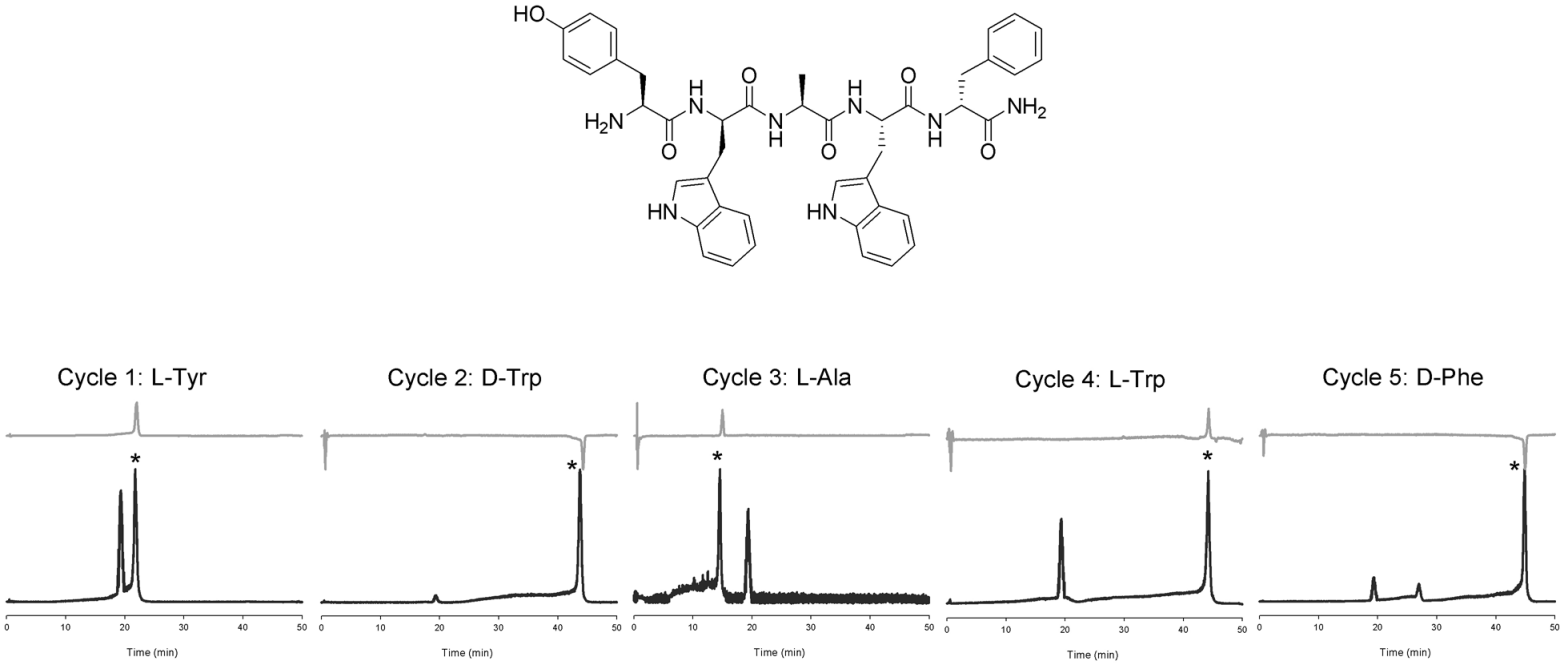
Ion chromatogram (dark) and CD absorption chromatogram (light) of the sequential Edman degradation products (asterisked) of a commercial pentapeptide. The unit of CD absorption is mdeg.

A commercial synthetic pentapeptide, l-Tyr-d-Trp-l-Ala-l-Trp-d-Phe amide (200 µg, 0.26 µmol), was subjected to the repeated Edman reaction, and the sequentially liberated PTH products were analyzed by RP-HPLC–MS/CD, as described above. Figure 2 shows our experimental sequence and configuration analysis data, which are identical to the information from the supplier. Several byproducts were detected through RP-HPLC; however, the major byproduct, diethoxy(phenylamino)methanethiol, was well separated from the PTH derivatives.

### Sequence and configuration analysis of halicylindramide C

**Figure 3:**
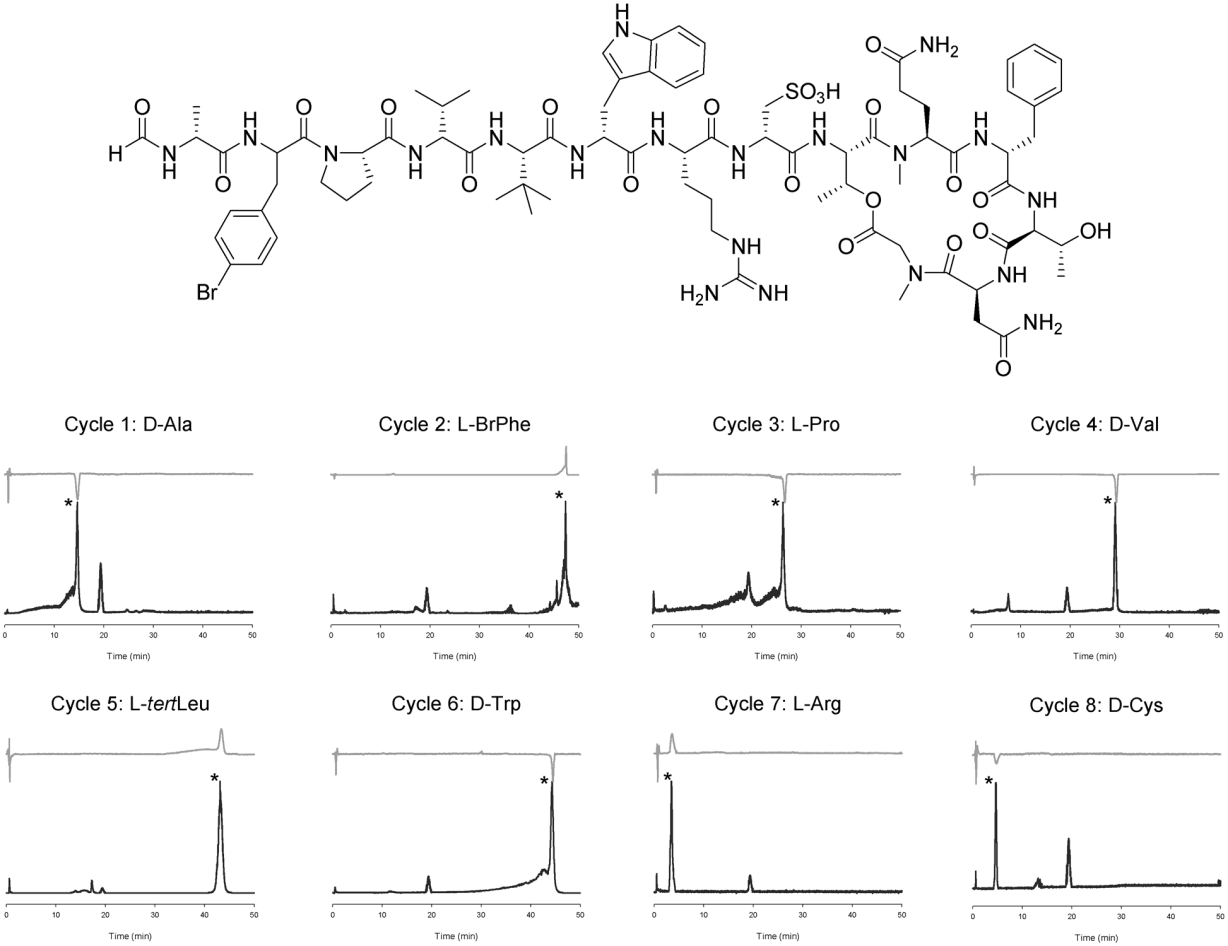
Ion chromatogram (dark) and CD absorption chromatogram (light) of the Edman degradation products (asterisked) of halicylindramide C. The unit of CD absorption is mdeg.

**Figure 4:**
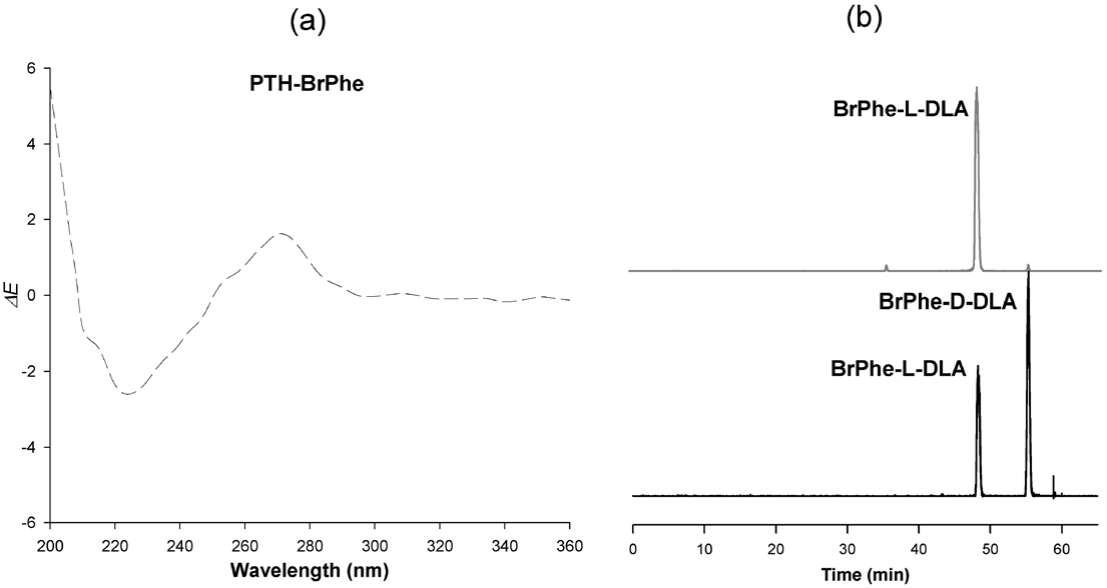
CD spectrum of the PTH derivative of BrPhe and ion chromatograms of DLA derivatives. (**a**) ECD spectrum of PTH-BrPhe derived from halicylindramide C. (**b**) The confirmation of absolute configuration of BrPhe in halicylindramide C by using the advanced Marfey’s method.

Halicylindramide C, a natural depsipeptide, was obtained from sponge with its N*-*terminus protected with a formyl group. Halicylindramide was first deformylated by the incubation of the peptide (200 µg, 0.11 µmol) with 500 µL of 2 N HCl at room temperature for 6 h. The deformylated halicylindramide C was then subjected to Edman reaction cycles, and the sequentially released PTH derivatives were analyzed by HPLC–MS/CD, as described above. Figure 3 shows the sequence and configuration analysis data of halicylindramide C. Its penultimate amino acid from the peptide N*-*terminus is BrPhe, which is not commercially available; the second Edman degradation cycle on halicylindramide C gave PTH-BrPhe, which showed two ion peaks at *m/z* values of 361 and 363 in the ion chromatogram and exhibited positive CD absorption at 270 nm in the CD spectrum. The CD spectrum of PTH-BrPhe (Fig. 4a) was similar to that of PTH-l-Phe. The absolute configuration of BrPhe was, therefore, determined to be l, which was confirmed using the advanced Marfey’s method (Fig. 4b, Table SI1).

Compared to the HPLC–MS signals of PTH-amino acids from cycles 1–8, the signals of the derivatives from cycle 9 were very weak, and their optical activities were not detectable on a CD-2095 detector. The weak signals might be attributed to the accumulation of excess PITC reagent unreacted and reaction byproducts from repeated cycles of Edman degradation. One of the challenges of manual Edman sequencing is the loss of parent peptide, which is caused by incomplete reaction frequently occurred at each cycle. It has been reported that the reaction yield of a gas-phase automated sequencer is extremely high, and a protein containing more than 90 amino acid residues was successfully analyzed by using such a sequencer by Hunkapiller and Hood^50,51^. The amino acid sequence and configuration analysis in longer peptides can be completed by the application of a HPLC–MS/CD system coupled to an automated gas-phase peptide sequencer.

### Configuration analysis of acid hydrolysate of peptide

**Figure 5:**
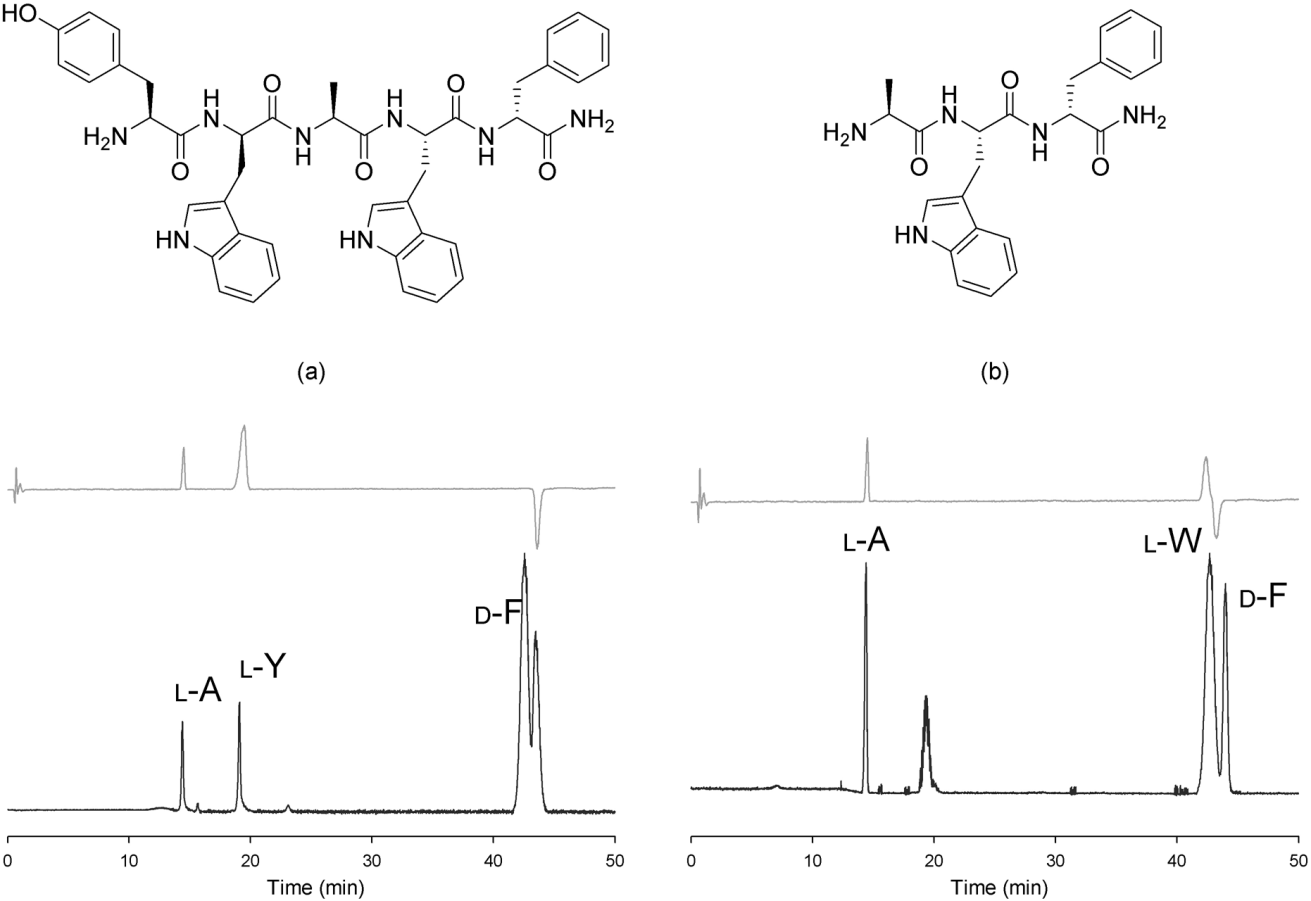
Ion chromatogram (lower) and CD absorption chromatogram (upper) of the PTH derivatives of acid hydrolysates of (**a**) the commercial pentapeptide l-Tyr-d-Trp-l-Ala-l-Trp-d-Phe and (**b**) the residual peptide l-Ala-l-Trp-d-Phe after elimination of l-Tyr and d-Trp by Edman reactions. The unit of CD absorption is mdeg.

HPLC–MS/CD was also applied to analyze the Edman reaction products of acid hydrolysate from a commercial pentapeptide containing both l-Trp and d-Trp. In this case Ala, Tyr, Phe, and Trp were detected by HPLC–MS, and their absolute configurations (except for Trp) were successfully determined by CD-2095. The Trp peak was observed in the electrospray ionization mass spectrometry (ESI–MS) chromatogram without CD absorption (Fig. 5a). Two cycles of the Edman reaction eliminated 1-Tyr and 2-Trp from the pentapeptide. The residual tripeptide, containing one Trp, was acid hydrolyzed; the hydrolysates were transformed to PTH derivatives using Edman reagent. When the derivatized hydrolysates were again analyzed by HPLC–MS/CD, l-Ala, l-Trp, and d-Phe were observed in the chromatogram (Fig. 5b). The configuration of 2-Trp was observed to be d.

**Figure 6:**
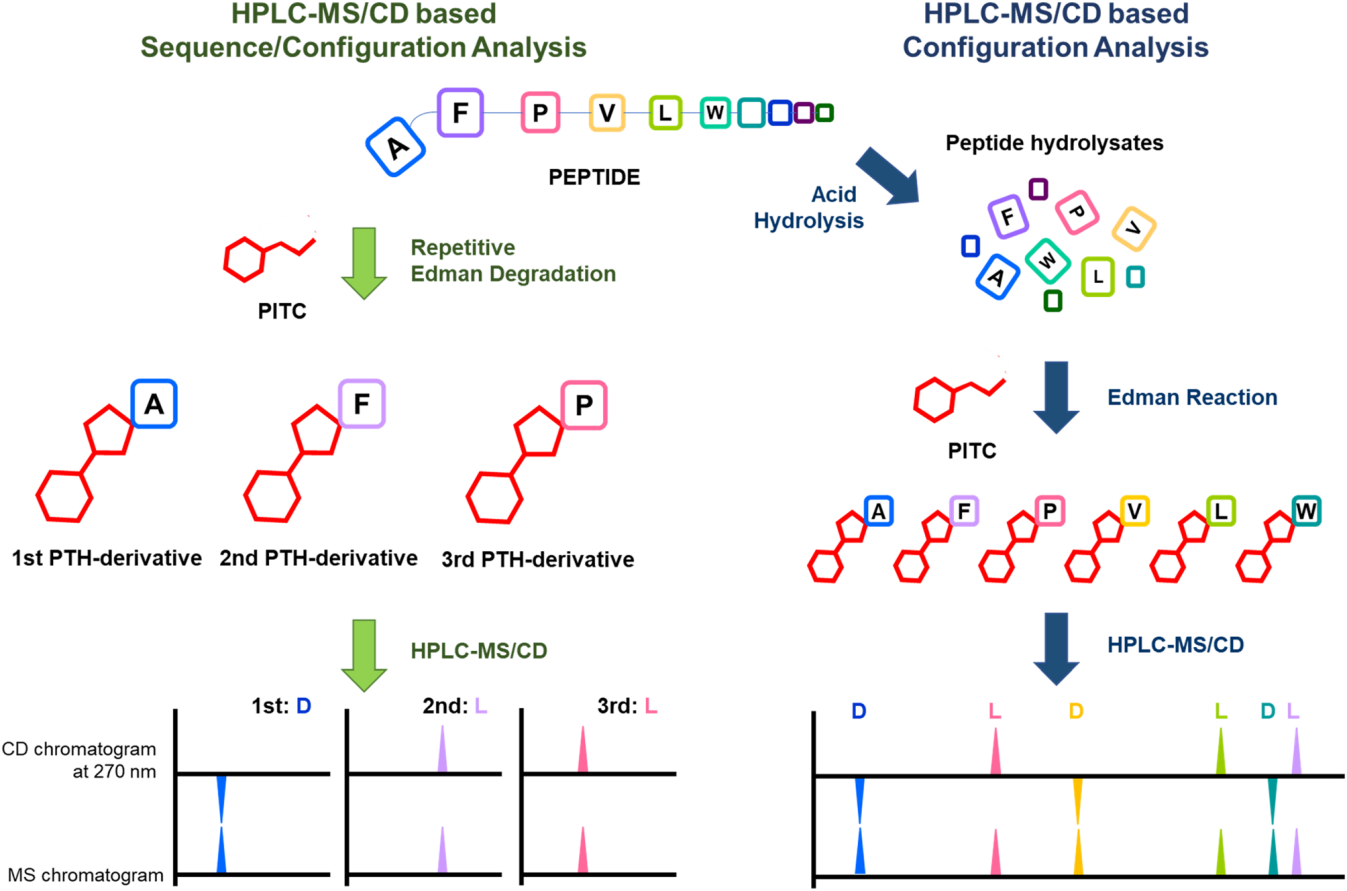
The general workflow of HPLC–MS/CD-based detection of liberated N-terminus phenylthiohydantoin amino acids method.

In conclusion, a method for the simultaneous determination of the sequence and absolute configuration of peptide amino acids by using a combination of the Edman procedure and HPLC–MS/CD is demonstrated. Amino acid configurations were determined by the CD spectral analysis of the sequential PTH-amino acids produced by the Edman reaction (Fig. 6). Because the analysis of the amino acid absolute configurations is based on spectroscopic characteristics, and not chromatographic behavior, this method can be extensively applied to unusual or novel amino acids (such as BrPhe in halicylindramide C) without the synthesis of standards. Furthermore, the combination of the gas-phase automated peptide sequencer and the HPLC–MS/CD system may provide a method to analyze longer peptides.

## Experimental section

### Chemicals

Halicylindramide C, formyl-d-Ala-l-BrPhe-l-Pro-d-Val-l-*tert*Leu-d-Trp-l-Arg-d-Cys(O_3_H)-cyclo(l-Thr-l-*N*MeGln-d-Phe-l-Thr-l-Asn-Sar), was isolated from *Halichondria* sp. and the structure was confirmed by MS and NMR experiments and the advanced Marfey’s method. In this study, we used the following commercial materials: sequencer-grade pyridine and sequencer-grade PITC (Wako Pure Chemical Industries, Osaka, Japan); boron trifluoride diethyl etherate complex (BF_3_–Et_2_O) (Tokyo Chemical Industry, Tokyo, Japan); HPLC-grade MeOH, EtOH, water, EtOAc, and acetonitrile (ACN, J. T. Baker, Pittsburgh, PA, USA); HCl (A. C. S. reagent grade, Sigma–Aldrich, St. Louis, MO, USA); heptane, anhydrous, 99% (Sigma–Aldrich Korea Ltd.); pentapeptide l-Tyr-d-Trp-l-Ala-l-Trp-d-Phe amide (Sigma–Aldrich).

### CD spectrometer

CD spectra were recorded between 200 and 360 nm using a JASCO J-715 system (JASCO, Tokyo, Japan). The cell length and volume were 1 cm and 200 µL, respectively. The scan speed and resolution were 50 nm/min and 0.5 nm, respectively.

### HPLC–MS/CD system

A Finnigan Surveyor Plus system (Thermo Fisher Scientific, Waltham MA, USA) consisting of a Finnigan Surveyor Autosampler Plus, an MS Pump Plus, a PDA Plus detector, a Thermo LTQ ESI MS system (Thermo Fisher Scientific), and a CD-2095 Plus Chiral Detector (JASCO) was used.

### Preparation of standard PTH-amino acids by Edman degradation

#### Coupling step

Amino acid was dissolved in 50% aqueous pyridine (10 mM). Next, 100 µL of the solution was mixed with 0.4 µL coupling solution (PITC/EtOH 1:2). The amino acid–coupling solution mixture was vortexed and heated at 50 °C for 30 min. After the coupling reaction, the mixture was dried in a centrifugal evaporator at 50 °C for 5 min. The dried residue was resuspended in 100 µL of deionized water and washed thrice with 100 µL of heptane/ethyl acetate (7:1, v/v). The aqueous layer was dried in a centrifugal evaporator at 50 °C for 15 min.

#### Cyclization/cleavage step

For the cyclization and cleavage reaction, 8 mM BF_3_–Et_2_O in ACN (100 µL) was added to the residue and the reaction mixture was vortexed and heated at 50 °C for 5 min. The reaction mixtures were dried under a stream of N_2_ gas. The dried residue was resuspended in 100 µL of water and washed thrice with 100 µL of heptane/ethyl acetate (1:5, v/v). The organic layer was dried under a stream of N_2_ gas.

#### Conversion step

The dried residue was mixed with 30 µL of 10 M HCl/MeOH at 50 °C for 30 min. The reaction mixtures were dried by centrifugal evaporation at 50 °C for 30 min.

#### Separation of PTH-amino acids via HPLC–MS/CD

PTH-amino acids were separated on an RP-HPLC column (Capcell Pak C-8 UGII column, 10 × 150 mm, 5.0 μm, Shiseido, Japan) with step gradient elution of 0.1% aqueous formic acid (eluent A) and 100% ACN (eluent B). Gradient program: 0–10 min, 4% B; 10–13 min, 4–15% B; 13–25 min, 15% B; 25–47 min, 17% B; flow rate, 1.0 mL/min. The column temperature was maintained at 30 °C. PTH-amino acids were detected through ESI–MS under the following conditions: sheath gas flow rate, 20 arb; aux gas flow rate, 10 arb; sweep gas flow rate, 1.5 arb; capillary voltage, 36 V; capillary temperature, 277 °C; tube lens voltage, 110 V.

#### Sequence and configuration analysis of commercial peptide and halicylindramide C

The commercial peptide, l-Tyr-d-Trp-l-Ala-l-Trp-d-Phe amide (200 µg, 0.26 µmol), was degraded by Edman degradation, as described above. After the cyclization/cleavage step, the residual peptide was dissolved in the aqueous layer. The aqueous layer was dried by centrifugal evaporation at 50 °C for 30 min. The dried residual peptide was subjected to the coupling step of the next cycle. The organic layer was subjected to the conversion step, and the final products were analyzed through HPLC–MS/CD. Halicylindramide C (200 µg, 0.11 µmol) was dissolved in 500 µL of 2 N HCl and deformylated at room temperature for 6 h. The Edman reaction was repeatedly performed on the deformylated halicylindramide C, as described above. PTH-amino acids, the Edman reaction product, were analyzed through HPLC–MS/CD.

#### Confirmation of absolute configuration of halicylindramide C by advanced Marfey’s method via HPLC–MS

Halicylindramide C (200 μg) was treated with 200 µL of 6 N HCl for 1 h at 110 °C. The hydrolysate was lyophilized, and the residue was dissolved in 100 µL of water and dried again. The dried hydrolysate was dissolved in 100 µL of 1 M NaHCO_3_ and separated into two vials. l-1-fluoro-2,4-dinitrophenyl-5-l-leucine amide (FDLA, 1%, 25 μL) in acetone was added to one vial and 25 μL of 1% d- and l-FDLA mixture in acetone to the other vial. The mixtures were vortexed and incubated at 40 °C for 60 min. After the reaction was quenched by the addition of 25 μL of 2 N HCl, the reaction mixture was diluted with 100 μL of methanol, and 10 μL of each solution was analyzed through HPLC–MS.

The reaction mixtures were separated on an RP-HPLC column (Capcell Pak C-18 MGII column, 10 × 150, 5.0 μm, Shiseido, Japan) with step gradient elution of 0.1% aq. TFA (eluent A) and 100% ACN (eluent B). Gradient program: 0–10 min, 30% B; 10–50 min, 30–70% B; 50–60 min, 70% B; 60–75 min, 70–100% B; 75–85 min, 100% B; flow rate, 100 μL/min. The column temperature was maintained at 30 °C. FDLA-amino acids were detected through ESI–MS under the following conditions: sheath gas flow rate, 14 arb; auxiliary gas flow rate, 10 arb; sweep gas flow rate, 1 arb; capillary voltage, 35 V; capillary temperature, 275 °C; tube lens voltage, 110 V.

#### Configuration analysis of peptide acid hydrolysate

A commercial pentapeptide (200 µg) was hydrolyzed by heating with 200 µL of 6 N HCl for 1 h at 110 °C. The acid hydrolysates were dried by centrifugal evaporation at room temperature for 1 h. The dried hydrolysates were dissolved in 100 µL of 50% aqueous pyridine and allowed to react with 5 µL of coupling solution (PITC/EtOH 1:2). The mixture was subjected to Edman degradation, as described above. Following the conversion step, the mixture was dissolved in 100 µL of MeOH and 2 µL of the solution was injected into the HPLC–MS/CD system.

## Supplementary Information

Supplementary Information.
