# Supplementary material for: Determination of sequence and absolute configuration of peptide amino acids by HPLC–MS/CD-based detection of liberated N-terminus phenylthiohydantoin amino acids
Source: Sci Rep. 2022 Jun 18;12:10285. doi: 10.1038/s41598-022-14205-x (PMC9206679; doi:10.1038/s41598-022-14205-x)
Supplement: Supplementary file 1 — Supplementary Information. [file 41598_2022_14205_MOESM1_ESM.pdf]

Determination of sequence and absolute configuration of peptide amino acids by HPLC-MS/CD-based detection of liberated *N*-terminus phenylthiohydantoin amino acids

Dongyup Hahn [1], Weihong Wang [2,3], Hyukjae Choi [4], and Heonjoong Kang [2,3,5*]*

[1] School of Food Science and Biotechnology & Department of Integrative Biotechnology, Kyungpook National University, Daegu 41566, South Korea

[2] Laboratory of Marine Drugs, School of Earth and Environmental Sciences, Seoul National University, NS-80, Seoul 08826, South Korea

[3] Research Institute of Oceanography, Seoul National University, Seoul 08826, South Korea

[4] College of Pharmacy, Yeungnam University, Gyeongsan 38541, South Korea

[5] Interdisciplinary Graduate Program in Genetic Engineering, Seoul National University, NS-80, Seoul 08826, South Korea

[*]Corresponding authors

Hyukjae Choi, Ph. D.

College of Pharmacy, Yeungnam University, Gyeongsan 38541, South Korea

280 Daehak-ro, Gyeongsan-si, Gyeongsangbuk-do, South Korea

Tel.: +82-53-810-2824 E-mail: h5choi@yu.ac.kr

Heonjoong Kang, Ph. D.

Laboratory of Marine Drugs, School of Earth and Environmental Sciences, Seoul National University, NS-80, Seoul 08826, South Korea

1 Gwanak-ro, Gwanak-gu, Seoul, South Korea

Tel.: +82-2-880-5730 E-mail: hjkang@snu.ac.kr

Table of Contents

Figure SI1. Edman degradation reaction mechanism ……………………………………………...... 4

Figure SI2. Byproduct formation of Edman degradation and the observation of byproduct peak in HPLC-MS/CD chromatogram ……………………………………………………………………...... 5

Table SI1. Absolute configuration of halicylindramide C confirmed by advanced Marfey's method..... 6

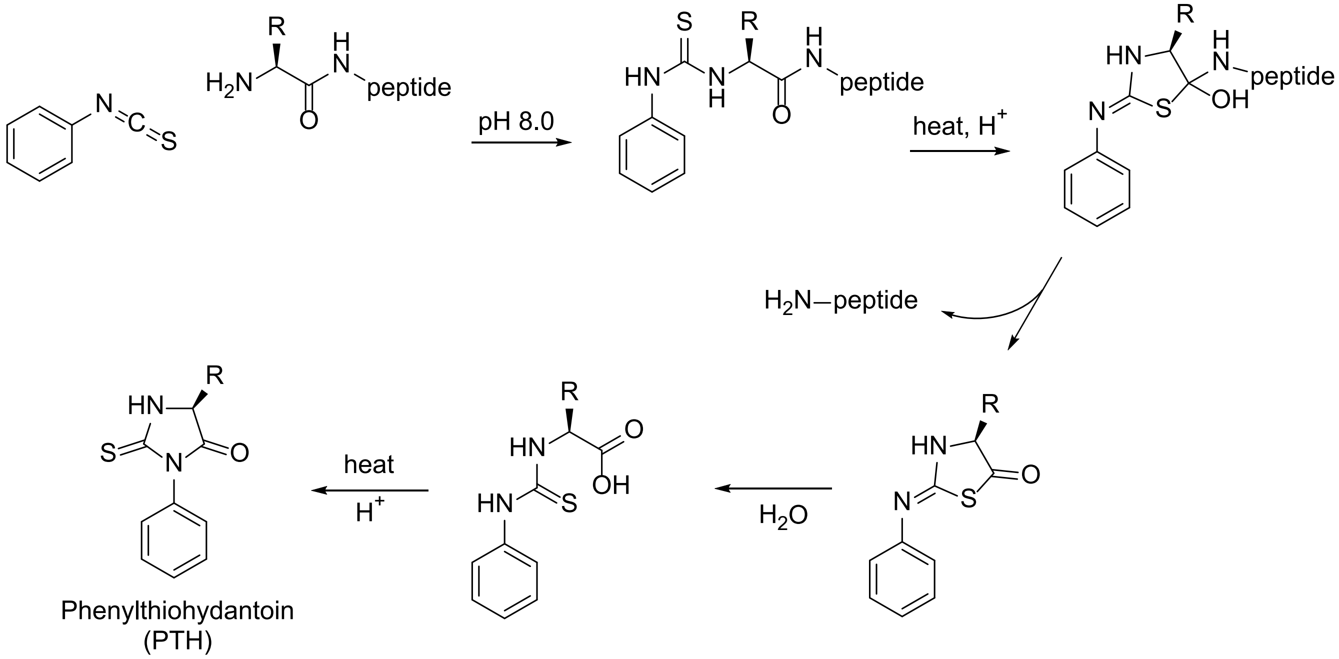

Figure SI1. Edman degradation reaction mechanism

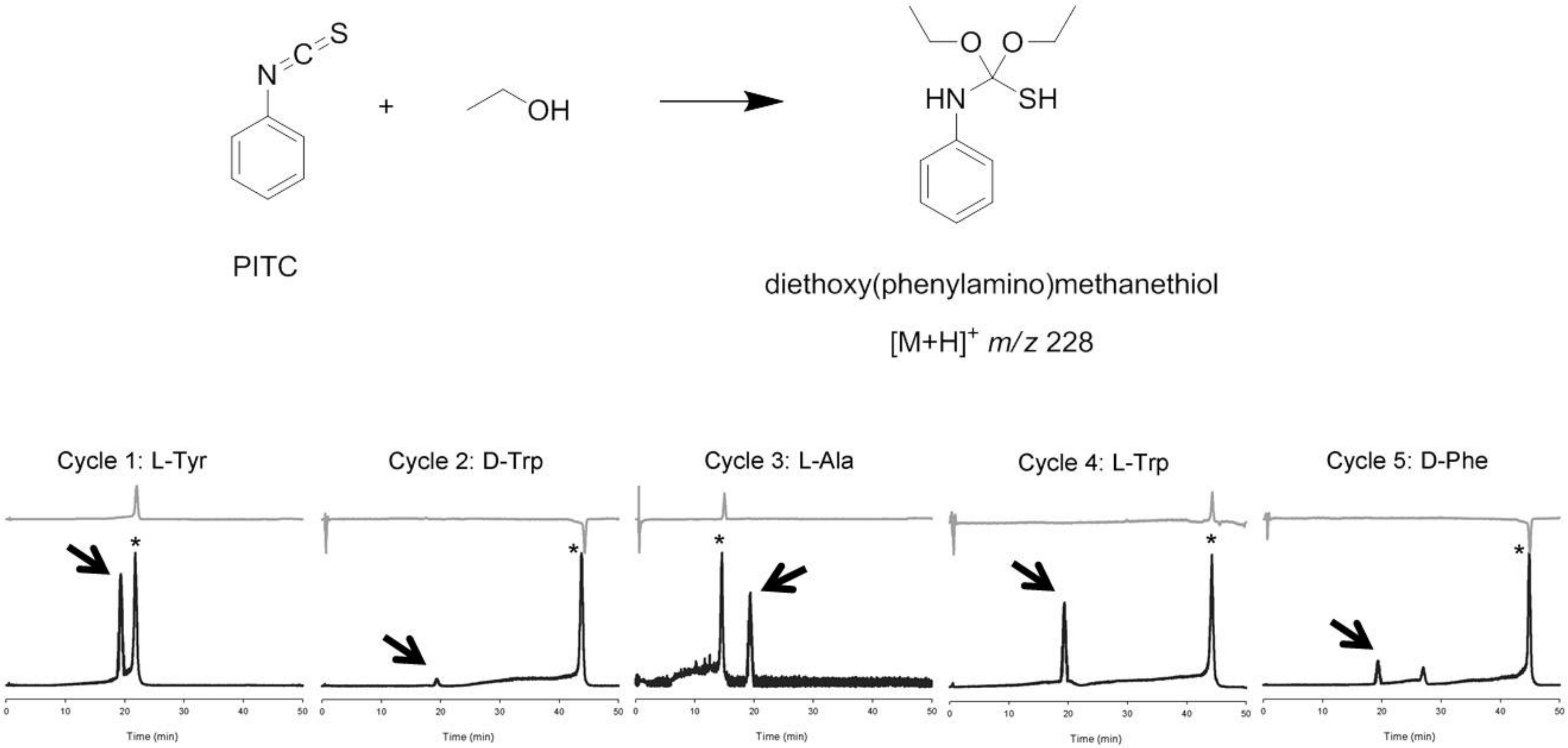

Figure SI2. Byproduct formation of Edman degradation and the observation of byproduct peak in HPLC-MS/CD chromatogram. Edman degradation products (asterisked) of a commercial pentapeptide and byproduct of Edman reaction (arrow)

Table SI1. Absolute configuration of halicylindramide C confirmed by advanced Marfey's method

Amino Acids	m/z range	Retention time of Amino acid-L-DLA (min)	Retention time of Amino acid-D-DLA (min)	Configuration assignment
1-Ala	495-496	38.40	34.49	D
2-BrPhe	648.5-649.5	48.32	55.27	L
3-Pro	521.5-522.5	33.71	37.28	L
4-Val	523-524	46.90	38.97	D
5-*t*Leu	537-538	41.42	50.39	L
6-Trp	610.5-611.5	46.25	41.42	D
7-Arg	694-695	25.98	21.32	L
8-Cys(HO_3)	532.5-533.5	11.66	12.74	D
9,12-Thr	525-526	27.73	33.90	L
10-NMeGln	567-568	28.75	29.69	L
11-Phe	457.5-458.8	43.50	49.38	D
13-Asn*	524.5-525.0	36.99	36.99	L

*The absolute configuration of Asn was determined through Marfey's method.
